# Supplementary material for: HNMT Upregulation Induces Cancer Stem Cell Formation and Confers Protection against Oxidative Stress through Interaction with HER2 in Non-Small-Cell Lung Cancer
Source: Int J Mol Sci. 2022 Jan 31;23(3):1663. doi: 10.3390/ijms23031663 (PMC8835856; doi:10.3390/ijms23031663)
Supplement: Supplementary file 1 [file ijms-23-01663-s001.zip › ijms-1544055-supplementary.pdf]

SUPPLEMENTARY INFORMATION

HNMT Upregulation Induces Cancer Stem Cell Formation and Confers Protection Against Oxidative Stress Through Interaction with HER2 in Non–Small-Cell Lung Cancer

Kuang-Tai Kuo[1,2], Cheng-Hsin Lin[3,4,5], Chun-Hua Wang[6,7], Narpati Wesa Pikatan[8], Vijesh Kumar Yadav[9], Iat-Hang Fong[9], Chi-Tai Yeh [9,10*], Wei-Hwa Lee[11], Wen-Chien Huang[12,13*]

[1] Division of Thoracic Surgery, Department of Surgery, School of Medicine, College of Medicine, Taipei Medical University, Taipei 110, Taiwan

[2] Division of Thoracic Surgery, Department of Surgery, Taipei Medical University—Shuang Ho Hospital, New Taipei City 235, Taiwan.

[3] Taipei Heart Institute, Taipei Medical University, Taipei, Taiwan; chlin99025@tmu.edu.tw

[4] Division of Cardiovascular Surgery, Department of Surgery, Shuang Ho Hospital, Taipei Medical University, New Taipei City, Taiwan.

[5] Division of Cardiovascular Surgery, Department of Surgery, School of Medicine, College of Medicine, Taipei Medical University, Taipei, Taiwan.

[6] Department of Dermatology, Taipei Tzu Chi Hospital, Buddhist Tzu Chi Medical Foundation,
New Taipei City, 231, Taiwan; 10205@s.tmu.edu.tw

[7] School of Medicine, Buddhist Tzu Chi University, Hualien 970, Taiwan

[8] Division of Urology, Department of Surgery, Faculty of Medicine, Universitas Gadjah Mada/Dr. Sardjito Hospital, Yogyakarta, 55281, Indonesia; narpatiwp@gmail.com

[9] Department of Medical Research & Education, Taipei Medical University—Shuang Ho Hospital, New Taipei City 235, Taiwan; ctyeh@s.tmu.edu.tw

[10] Department of Medical Laboratory Science and Biotechnology, Yuanpei University of Medical Technology, Hsinchu 300, Taiwan

[11] Department of Pathology, Taipei Medical University—Shuang Ho Hospital, New Taipei City 235, Taiwan; whlpath97616@s.tmu.edu.tw

[12] Department of Medicine, MacKay Medical College, New Taipei City 252, Taiwan; wjhuang0@gmail.com

[13] Division of Thoracic Surgery, Department of Surgery, MacKay Memorial Hospital, Taipei 104, Taiwan

Correspondence: Corresponding Author Chi-Tai Yeh, MD., PhD, Department of Medical Research, Taipei Medical University-Shuang Ho Hospital, New Taipei City, Taiwan; Department of Pathology, Taipei Medical University-Shuang Ho Hospital, Taipei, Taiwan. Phone: 886-2-2490088 ext. 8881; FAX: 886-2-2248-0900 E-mail: ctyeh@s.tmu.edu.tw; Wen-Chien Huang, MD., Ph.D, Division of Thoracic Surgery, Department of Surgery, MacKay Memorial Hospital, Taipei 104, Taiwan; Phone: 886-2-2490088 ext. 2919. FAX: 886-2-2248-0900, E-mail: wjhuang0@gmail.com

Table S1: Primary antibodies of Western blots.

No.	Target	Dilution	MW (kDa)	Source	
1	HER2	1:1000	185	#4290	Cell Signaling
2	p-HER2	1:1000	185	#6942	Cell Signaling
3	HER3	1:1000	185	#12708	Cell Signaling
4	Vimentin	1:1000	57	#5741	Cell Signaling
5	N-Cadherin	1:1000	140	#13116	Cell Signaling
6	KLF4	1:1000	60	ab129473	Abcam
7	NANOG	1:1000	37	ab109250	Abcam
8	OCT4	1:1000	45	ab200834	Abcam
9	CD133	1:1000	110	ab222782	Abcam
10	Nrf2	1:1000	85	ab62352	Abcam
11	HO-1	1:1000	33	ab52947	Abcam
12	HNMT	1:1000	33	11874-1-AP	Thermofisher
13	β-actin	1:10000	42	66009-1-Ig	proteintect

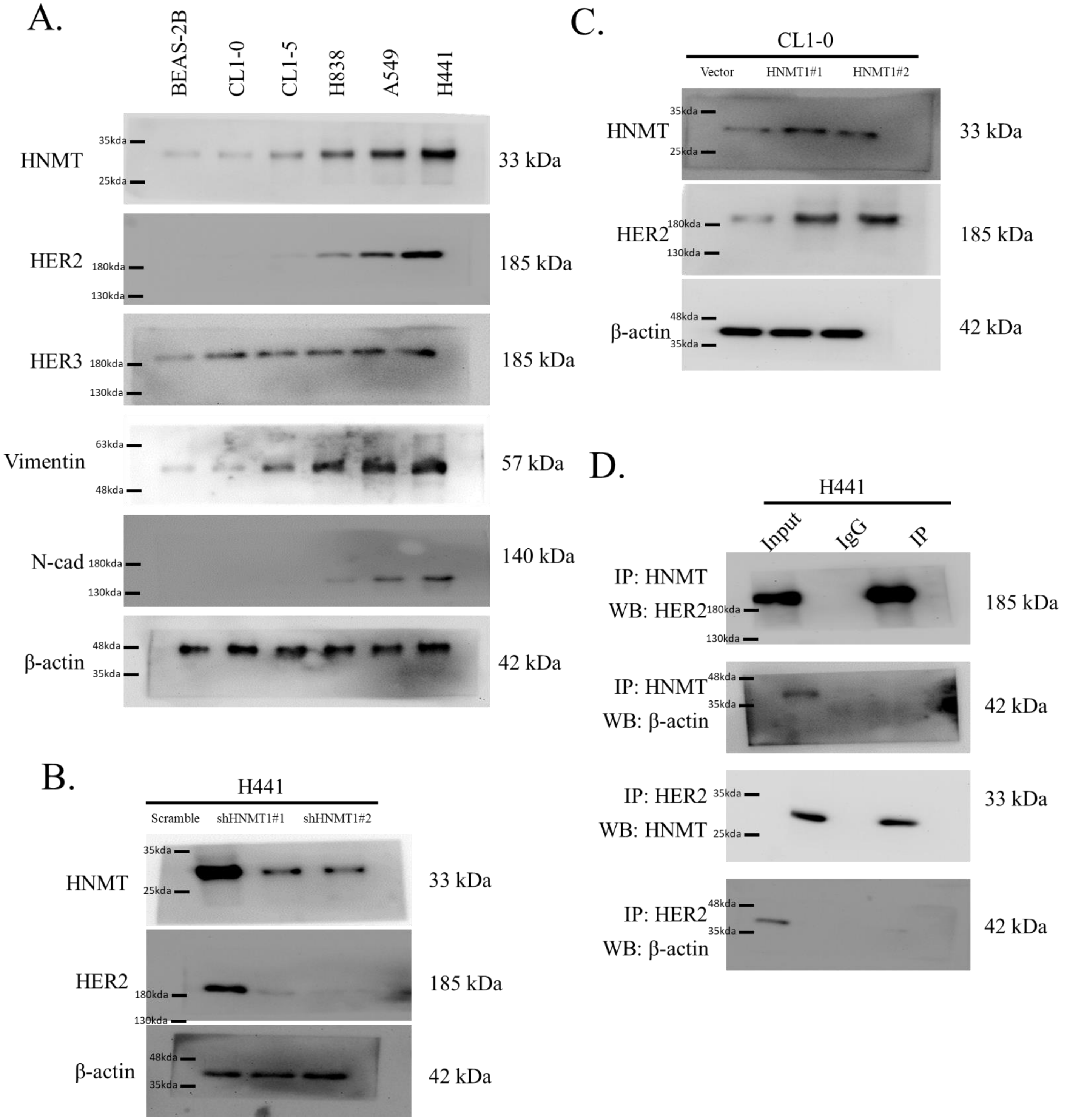

Supplementary Figure S1. Full-size blots of Figure 2. A, B, C and D

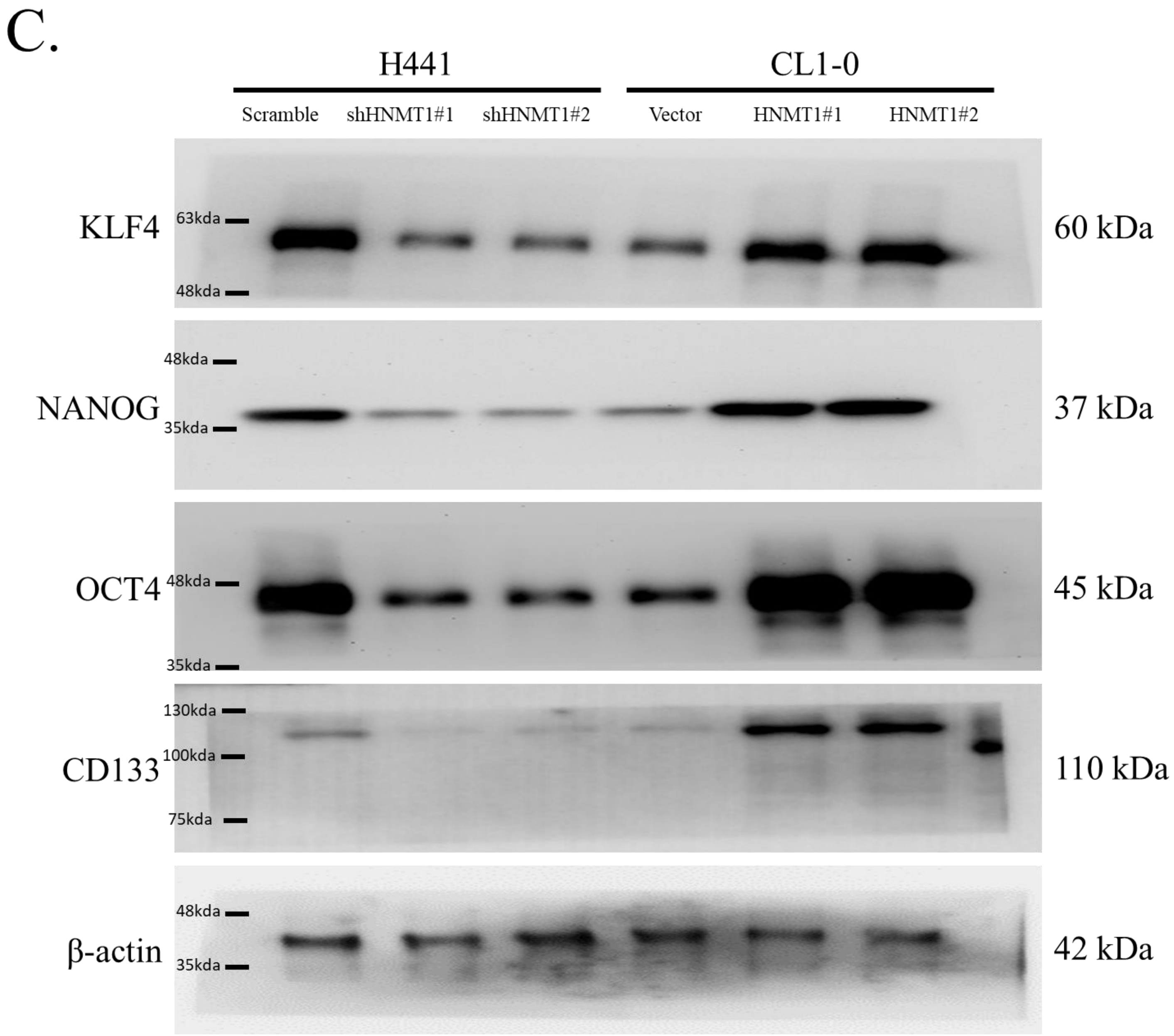

Supplementary Figure S2. Full-size blots of Figure 3C

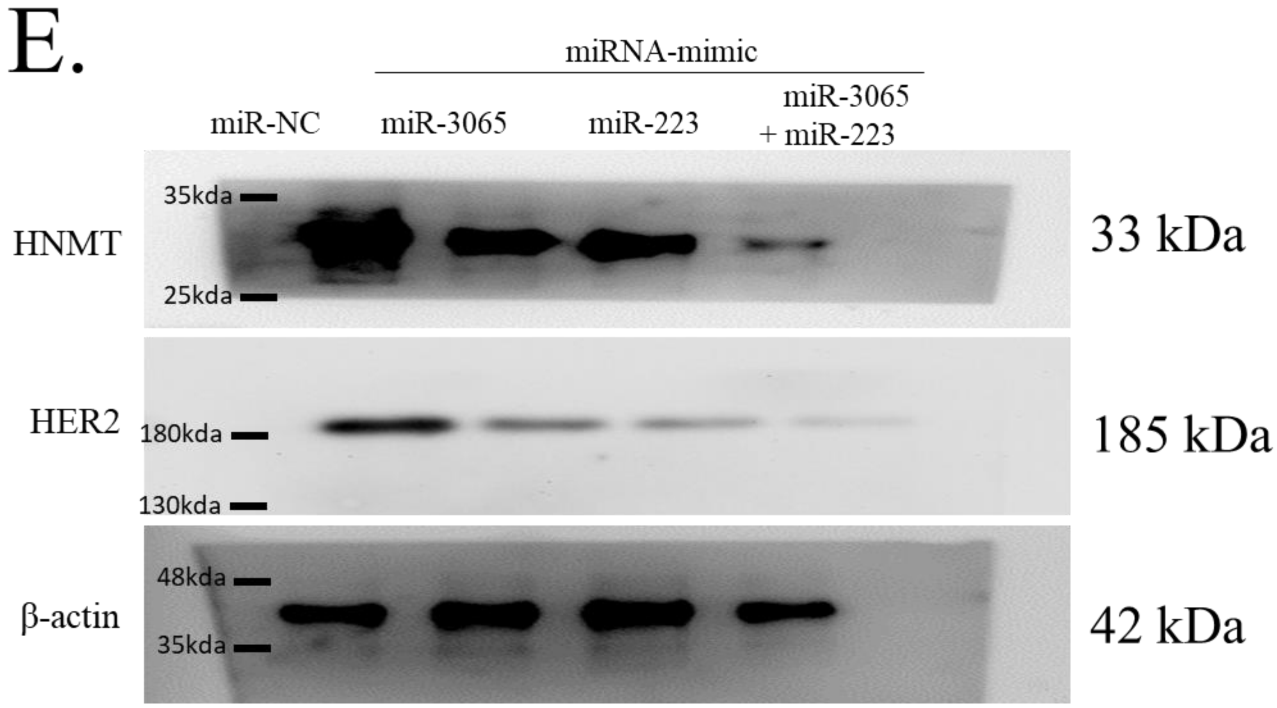

Supplementary Figure S3. Full-size blots of Figure 4E

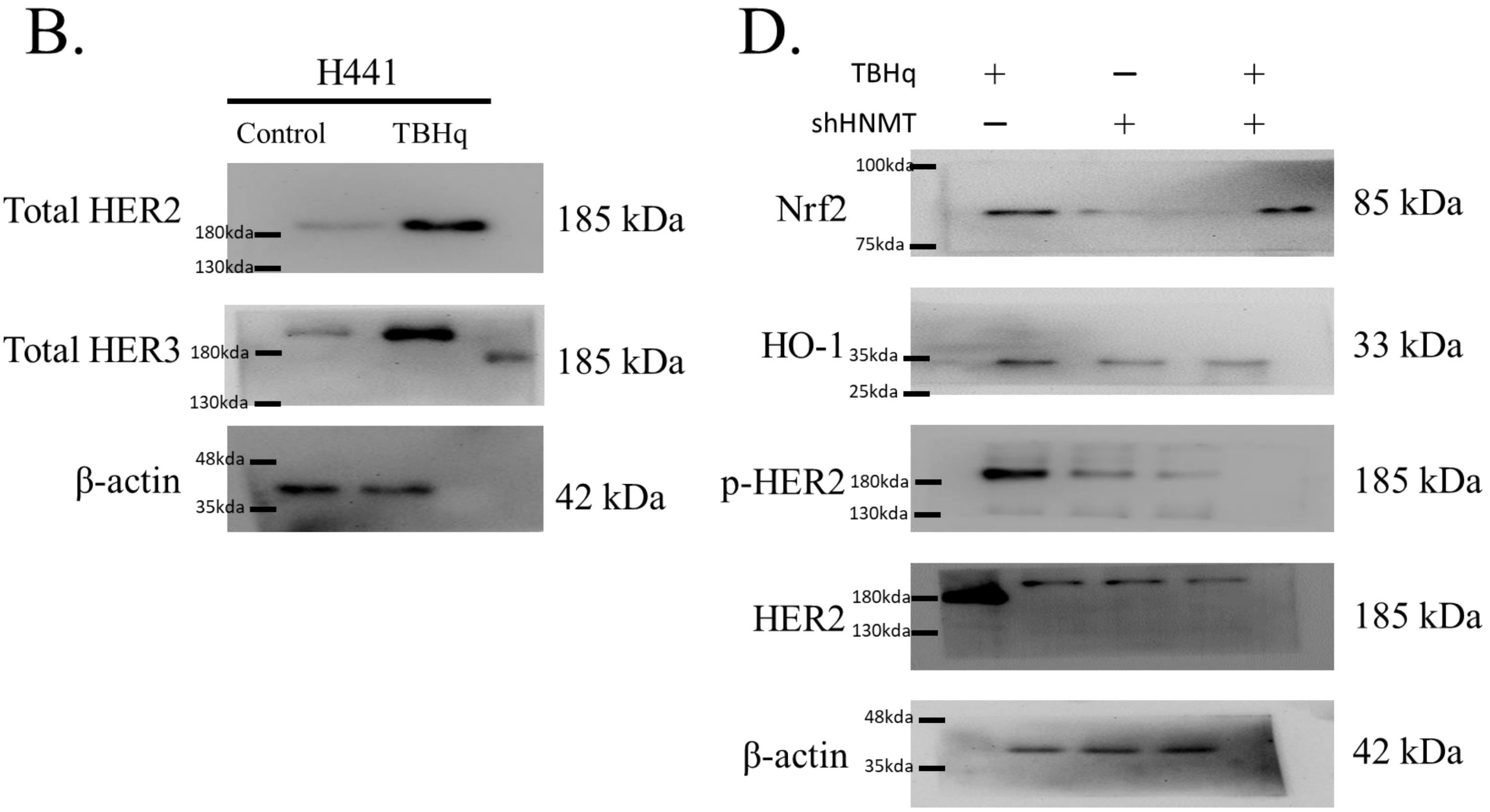

Supplementary Figure S4. Full-size blots of Figure 5B & 5D.
